# Analyzing the distribution of rabies clinics and achievements of standardized rabies clinics implementation in mainland China

**DOI:** 10.1186/s12913-019-4730-9

**Published:** 2019-12-11

**Authors:** Zhe Du, Qingjun Chen, Xinjun Lyu, Tianbing Wang, Chuanlin Wang

**Affiliations:** 1 Trauma Center, Peking University People’s Hospital, No.11 South Xizhimen Street, Beijing, 100044 China; 2 Department of Emergency, Beijing Hepingli Hospital, Beijing, China; 3 China Center for Disease Control and Prevention, Institute for Viral Disease Prevention and Control, Beijing, China; 4 Department of Emergency, Peking University People’s Hospital, Beijing, China

**Keywords:** Achievements, Rabies, Standardized clinics, Stratified sampling

## Abstract

**Background:**

For rabies prevention and treatment, the Chinese government has been establishing standardized rabies clinics since 2016. This study aimed to investigate the distribution of rabies clinics and the achievements of newly-implemented standardized rabies clinics in mainland China, for the purpose of providing further rabies control strategies.

**Methods:**

The number of rabies clinics, including per million inhabitants in each region, was determined. We sampled 1200 clinics from 8 provinces by multi-stage stratified sampling, and a questionnaire survey was carried out to record each clinic’s achievements. Data collected from 1185 questionnaires were analyzed.

**Results:**

We found that rabies clinics were mostly located in the southwest, central, and eastern regions of China; these accounted for 67.1% of all clinics. The eastern and south regions showed the lowest number of rabies clinics per million inhabitants (0.15 and 0.12, respectively). The total standard-reaching rate of rabies clinics in mainland China was only 11.0%, with significant differences in the rate among regions (*X*^2^ = 33.004, *p* <  0.001). Specifically, the qualified rates of supporting facilities and functional areas were 13.9% (*X*^2^ = 34.003, *p* <  0.001) and 56.1% (*X*^2^ = 9.943, *p* = 0.019), respectively. Vaccines with 2 different substrates and professional flushing equipment were provided by 40.5% (*X*^2^ = 27.935, *p* = 0.001) and 37.7% (*X*^2^ = 54.922, *p* = 0.001) of clinics, respectively.

**Conclusion:**

Regional differences do exist in the distribution of rabies clinics in mainland China, with relative low number per million population in south and eastern China. There are few standardized rabies clinics in mainland China. Efforts are needed to establish supporting facilities, especially for wound treatment and vaccination. Future research should focus on the improvement of rabies clinics standardization.

## Background

Rabies is a fatal and zoonotic disease transmitted to humans through a bite or scratch from an infected animal [1–3]. In unvaccinated humans, the fatality rate is almost 100% [1, 4] with approximately 59,000 human deaths occurring globally every year, 95% of which occur in Asia and Africa [3, 5]. However, rabies is preventable and the World Health Organization (WHO) has launched a proposal to eliminate its transmission from dogs to humans before 2030 [6]. Rabies has been controlled or even eliminated for decades in some areas of Asia, including Malaysia, Japan, and many island countries or regions [7], but China is a high-risk environment for this disease, with the second highest number of human cases after India [7–9]. Despite the major control achievements conducted in the past decade, rabies remains an important public health problem in China, where nearly 1000 human rabies cases were reported in 2014 [9–11].

Rabies can be prevented by standardized post-exposure prophylaxis (PEP), an important measure recommended by the WHO that consists of appropriate wound treatment followed by completion of the rabies vaccination series and administration of rabies immunoglobulin (RIG) when warranted [3]. To end human deaths due to rabies by 2030, PEP must be strictly implemented [12]. China’s national policy requires wound treatment and PEP vaccination for category II and III exposures, in addition to RIG administration for category III [3, 13]. For the standardized treatment of rabies-exposed patients, the establishment of standardized rabies clinics, with both appropriate supporting facilities and qualified functional areas, is required. The qualified supporting facilities must meet certain achievement standards, including providing vaccines with 2 different substrates, rabies immunoglobulin, and first aid drugs (adrenaline, dopamine, atropine, etc.). The qualified functional areas in the clinics include waiting, reception, wound treatment, vaccination, and rescue areas. The Chinese government has been promoting the construction of standardized rabies clinics to accomplish the requirements of standardized PEP and subsequently reduce the incidence of rabies.

In the Philippines, the number of government-run animal bite treatment centers (ABTCs) has been steadily rising, and a total of 513 ABTCs had been established by 2017, with 70 out of 82 provinces having at least one ABTC [14]. However, there are very few studies regarding the distribution of rabies clinics in China. This study aimed to investigate the distribution of rabies clinics in mainland China and evaluate the achievements accomplished through their implementation, to promote further the establishment of standardized clinics for rabies control in China.

## Methods

### Investigation method

**Fig. 1:**
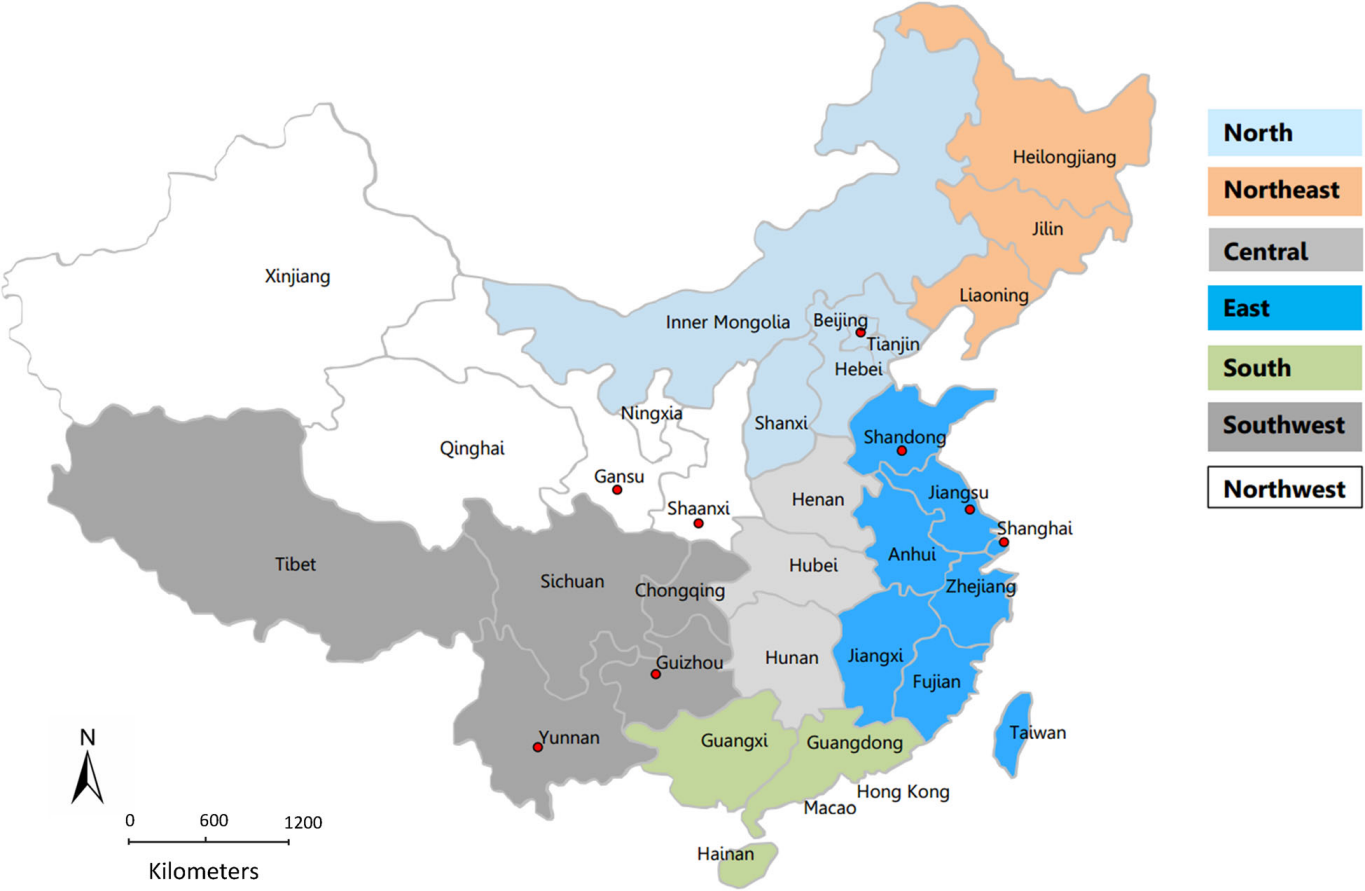
Seven geographical regions of midland China, used for data analysis in this study. The map depicted in figure (1) is our own, not taken from another source. The software (Photoshop CS5, Adobe Systems, USA) is used in depicting figure (1)

The 2017 data of rabies clinics in the provinces of mainland China were obtained from the database of the National Disease Reporting Information System (NDRIS) of the Chinese Center for Disease Control and Prevention (China CDC). The population of each province in 2017 was published by the National Statistical Bureau. Two of the researchers (ZD and QJC) participated in the data extraction, and performed data checks, and cleaning in order to ensure data quality. These data were used to analyze the distribution of rabies clinics and the number of clinics per million population in mainland China. The number of rabies clinics was determined in 7 geographical regions (except Hong Kong, Macao, and Taiwan) (Fig. 1). The number of clinics per million population was obtained by dividing the number of clinics in each region by the respective region population.

Multi-stage stratified random sampling was applied. In the first stage of sampling, the total number of rabies clinics in all provinces and municipalities was stratified according to the number published by the China CDC in 2017, and each level was randomly sampled. The total number of clinics was then divided into 4 layers. Among 8 China provinces and municipalities, Shanghai and Beijing, Guizhou and Yunnan, Shaanxi and Gansu, and Shandong and Jiangsu were defined as regions 1 (metropolis), 2 (southwest), 3 (northwest), and 4 (eastern), respectively (Fig. 1). From the perspective of topography, climate, population distribution, and economy, China is divided into 7 regions. The 6 provinces selected for this study are distributed in only 3 of the 7 regions (southwest, northwest, and eastern), and the 2 municipalities (metropolis) were listed separately. In the second stage of sampling, 5 municipal districts or prefecture-level cities were screened from provinces or municipalities in equal distance according to the sampling method.

### Evaluation of standardized rabies clinics achievements

A questionnaire designed to evaluate rabies clinics was conducted in 40 municipal districts or prefecture-level municipalities. Evaluation of standardized rabies clinics was carried out from 2 perspectives. We first analyzed the qualified supporting facilities considering: (1) being open for 24 h; (2) providing 2 different matrix vaccines, including the human diploid cell rabies vaccine; (3) providing human rabies immunoglobulin or anti-rabies serum at 24 h; (4) providing tetanus immunoglobulin, tetanus vaccine, or other essential drugs to prevent tetanus; (5) being equipped with professional washers and flushing fluids; (6) having surgery conditions and the ability to deal with complex wounds; (7) being equipped with an information reporting system; and (8) being equipped with disinfection and first aid drugs, such as 20% soap water, 2–3% iodine wine, or 75% alcohol for wound cleaning and disinfection, as well as with adrenaline and other first aid drugs (including promethazine hydrochloride, dexamethasone, cedilanid, m-hydroxylamine bistartrate, lobeline, nicosamide, or calcium gluconate injection). Second, the qualified functional areas were also evaluated, including: (1) waiting and observation; (2) reception (receiving a patient for treatment); (3) wound treatment; (4) vaccination; and (5) rescue areas (the area where critical patients were treated). All rabies clinics that met the above requirements were regarded as standardized rabies clinics and included in this study.

### Statistical analysis

Initial data were collected and analyzed with SPSS 20.0 (IBM, Armonk, NY). Achievements of standardized rabies clinics, including qualified supporting facilities and functional areas among different regions, were compared. Descriptive analysis and Chi–square test were used for statistical analysis. *P*-values of < 0.05 were considered statistically significant.

## Results

**Fig. 2:**
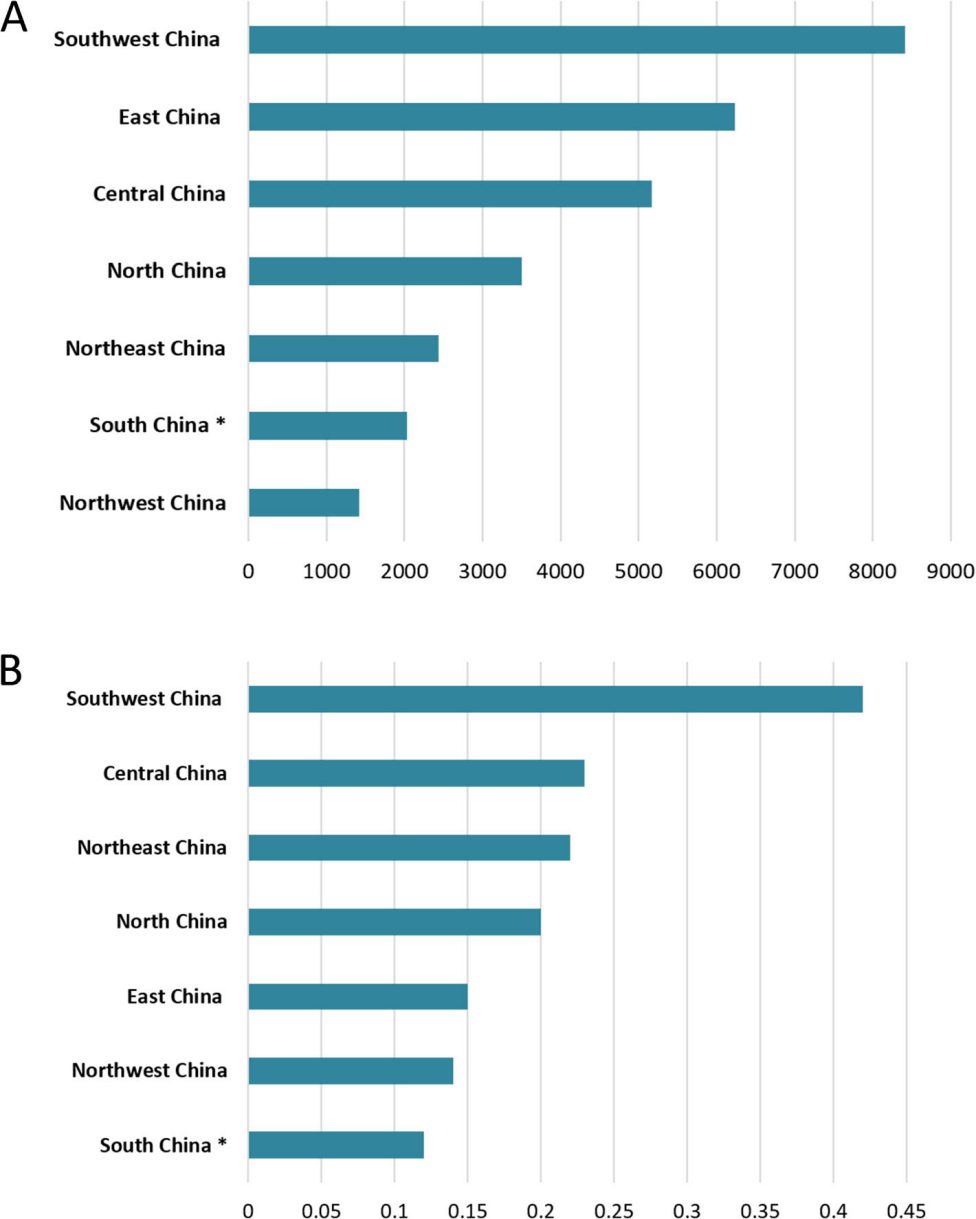
Distribution of rabies clinics in mainland China. **a** Number of outpatient clinics in 7 specific geographical regions. **b** Number of rabies clinics per million inhabitants in each region. * the data of distribution of rabies clinics in Hong Kong, Macao, and Taiwan are not collected

According to the NDRIS, 29106 rabies clinics from all midland China provinces (excluding Hong Kong, Macao, and Taiwan) were recorded. The 3 regions with the highest number of rabies clinics by the end of 2017 were the southwest (8318), eastern (6231) and central (5163) regions, accounting for 67.1% of the total number of clinics (Fig. 2a). There were only 1415 rabies clinics in northwest China, which does not correlate with its vast area (Fig. 2a). The 3 regions with the highest number of clinics per million inhabitants were the southwest (0.42), central (0.23), and northeast China (0.22); the eastern and south regions being the lowest (0.15 and 0.12, respectively) (Fig. 2b).

A total of 1185 questionnaires from 1200 clinics were collected. The achievements of rabies clinics were evaluated in 2 aspects: qualified supporting facilities and functional areas. Table 1 shows the standard-reaching rates of standardized rabies clinics (11.0%), qualified supporting facilities (13.9%), and qualified standard functional areas (56.1%) in mainland China, with significant differences among different regions (*X*^2^ = 33.004, *p* < 0.001; *X*^2^ = 34.003, *p* < 0.001; *X*^2^ = 9.943, *p* = 0.019; respectively).

**Table 1:** Achievements of standardized rabies clinics, including qualified supporting facilities and functional areas, in mainland China and respective regions

	Standardized rabies clinics	Qualified supporting facilities	Qualified functional areas
Region 1 (n, %)	30 (18.8)	55 (34.4)	55 (34.4)
Region 2 (n, %)	20 (3.8)	20 (3.8)	280 (53.8)
Region 3 (n, %)	20 (7.0)	20 (7.0)	170 (59.7)
Region 4 (n, %)	60 (27.3)	70 (31.8)	160 (72.7)
Total (n, %)	130 (11.0)	165 (13.9)	665 (56.1)
*X*^*2*^*, P value*	33.004, < 0.001	34.003, < 0.001	9.943, 0.019

Region 1: Beijing and Shanghai; Region 2: Guizhou and Yunnan; Region 3: Shaanxi and Gansu; Region 4: Shandong and Jiangsu. The percentage (%) = number of rabies clinics / total clinics

The reaching-rate of several achievements in all rabies clinics was approximately 70% or higher, with the exception of provision of professional flushing equipment (37.7%), vaccines with 2 different substrates (including human diploid cell rabies vaccine) for 24 h (40.5%), and tetanus immunoglobulin (59.5%) (Table 2). Some achievements in specific regions were lower than those in all of mainland China. For example, in region 2 (Guizhou and Yunnan), 51.0% of the supporting facilities were opened for 24 h (compared to 69.6% for all regions). Similarly, in region 3 (Shaanxi and Gansu), 54.4% of the supporting facilities were equipped with information reporting systems (compared to 71.6% for all regions).

**Table 2:** Achievements of qualified supporting facilities in rabies clinics in mainland China and respective regions

	Region 1	Region 2	Region 3	Region 4	Total	*X*^*2*^*, P value*
Open 24 h (n, %)	160 (100)	265 (51.0)	245 (86.0)	155 (70.5)	825 (69.6)	38.297, 0.001
Provide vaccines with 2 different substrates (n, %)	90 (56.3)	195 (37.5)	50 (17.5)	145 (65.9)	480 (40.5)	27.935, 0.001
Provide rabies immunoglobin (n, %)	160 (100)	465 (89.4)	200 (70.2)	200 (90.9)	1025 (86.5)	19.439, 0.002
Provide tetanus immunoglobulin (n, %)	130 (81.3)	260 (50.0)	180 (63.2)	135 (61.4)	705 (59.5)	10.556, 0.014
Provide professional flushing equipment (n, %)	135 (84.4)	90 (17.5)	95 (33.3)	125 (56.8)	445 (37.7)	54.922, 0.001
Surgical conditions in the clinics (n, %)	160 (100)	455 (88.4)	230 (80.7)	190 (86.4)	1035 (87.7)	7.195, 0.066
Equipped with information reporting system (n, %)	160 (100)	345 (67.0)	155 (54.4)	185 (84.1)	845 (71.6)	25.457, < 0.001
Equipped with disinfection and first aid drugs (n, %)	155 (96.9)	390 (75.7)	250 (87.7)	195 (88.6)	990 (83.9)	10.426, 0.015

Region 1: Beijing and Shanghai; Region 2: Guizhou and Yunnan; Region 3: Shaanxi and Gansu; Region 4: Shandong and Jiangsu

n (%) = number of qualified rabies clinics (number of rabies clinics / total clinics)

The reaching-rate of vaccination area was 53.1% in region 1, which was lower than that in the other 3 regions (*X*^2^ = 20.633, *p* < 0.001). The standard-reaching rate of rescue area was 72.6%, which was the lowest rate of all achievements (Table 3).

**Table 3:** Achievements of qualified functional areas in rabies clinics in mainland China and respective regions

	Region 1	Region 2	Region 3	Region 4	Total	*X*^*2*^*, P value*
Waiting area (n, %)	135 (84.4)	415 (79.8)	245 (86.0)	220 (100)	1015 (85.7)	9.754, 0.021
Reception area (n, %)	160 (100)	485 (93.3)	255 (89.5)	220 (100)	1120 (94.5)	9.671, 0.055
Wound treatment area (n, %)	160 (100)	400 (75.0)	240 (84.2)	220 (100)	1020 (86.1)	19.119, < 0.001
Vaccination area (n, %)	85 (53.1)	515 (99.3)	225 (78.9)	220 (100)	1045 (88.2)	20.633, < 0.001
Rescue area (n, %)	155 (96.9)	305 (58.7)	240 (84.2)	220 (72.7)	860 (72.6)	22.744, < 0.001

Region 1: Beijing and Shanghai; Region 2: Guizhou and Yunnan; Region 3: Shaanxi and Gansu; Region 4: Shandong and Jiangsu

Percentage (%) = number of qualified rabies clinics / total clinics

Reception area: area where a patient is received for treatment; Rescue areas: area where critical patients are treated

## Discussion

The PEP vaccination rate for rabies among China’s general population has been reported as extremely low [3]. PEP is essential to rabies prevention after potential exposure. The Chinese government has promoted the establishment of standardized rabies clinics since 2016, with the aim of increasing access to PEP, enhance community awareness of rabies prevention methods, and eventually eliminate dog-mediated human rabies in China. This study investigated the distributions of rabies clinics in China, and investigated the reaching-rate of several proposed achievements in those clinics through a sampling survey.

We found that rabies clinics in China were mostly concentrated in the southwest, eastern, and central regions by the end of 2017. Rabies cases in China are primarily detected in rural areas of the southern and eastern provinces, and are reportedly rare in urban areas such as Beijing and Shanghai [15]. However, we identified only 2034 rabies clinics in southern China, ranking this region sixth out of the 7 regions analyzed. In addition, south China had the lowest number of outpatient clinics per million inhabitants. These findings indicate the need for increased government investment into the construction of rabies clinics in China’s southern regions. This is also necessary in the eastern region, where the number of clinics per million inhabitants was also low despite this region having a high rabies incidence [12]. The disproportional prevalence of rabies in rural areas [9] likely reflects the unequal distribution of medical resources in China [3]. Therefore, the Chinese government should increase the establishment of rabies clinics in remote rural areas of China, which would be particularly beneficial for high-risk subpopulations, especially children [1]. According to the literature, as of July 2017 there were 513 ABTCs in the Philippines. Across all provinces, there was a wide variation, with an average of 0.63 and a maximum of 3.15 ABTCs/100,000 population. Only 16 provinces have currently reached the target of 1 ABTC per 100,000 population [14]. However, in this study, there was an average of 2.11 and a maximum of 4.21 rabies clinics/100,000 population in China (Fig. 2b).

The qualified rate of standardized rabies clinics was relatively low (11.0%), mainly because the qualified rate of supporting facilities was only 13.9%. The clinics with the higher unqualified rate were concentrated in regions 2 and 3, which are the underdeveloped areas in northwest and southwest China. The main reasons for the unqualified support facilities was lack of professional flushing equipment (37.7%) and lack of vaccines with 2 different substrates (40.5%). The standard wound treatment regimen includes wound flushing and disinfection, as well as surgical procedures if necessary [13]. Flushing and disinfection are essential steps to reduce the rabies viral load and the risk of secondary bacterial infection within the wound site [3]. Moreover, to effectively control rabies, it is particularly important to provide an alternative vaccine in cases when the initially-implemented vaccine has failed. We identified insufficient coordination of 2 different matrix vaccines in standardized rabies clinics nationwide. Finally, we also found that in terms of functional areas in rabies clinics, the main problematic factor was the rescue area, which had a standard-reaching rate of 72.6%. This shows that the construction of standardized rescue areas in the clinics is insufficient and needs to be urgently addressed.

To control and eliminate rabies in China, an integrated intervention strategy based on WHO recommendations must be adopted; this will require the collective efforts of multiple sectors, including city management offices and public health, veterinary, and educational departments [16]. For example, in high-risk provinces, such as Guizhou, Guangxi, and Guangdong, the government recently included PEP fees, including the vaccine fees, into the social insurance system, which increased the rate of individuals who had been bitten and who had access to PEP compared to the rate observed in previous years [9]. The Chinese government has been actively supporting the construction and management of outpatient clinics aimed to prevent and treat rabies after exposure, establishing a standardized system, and implementing qualification certification to achieve the elimination of human rabies by 2030. However, the construction of standardized rabies clinics in key areas requires time, and relevant policies need to be considered to improve the eligibility rate of these clinics. An integrated “One Health” approach should be encouraged; the combined effort will contribute to the goal of eliminating human rabies [17].

One limitation of this study lies in the fact that only 8 provinces were studied and each province was further limited to 5 municipalities. In addition, there are differences in economic, social development, and cognitive levels among provinces, cities, and municipalities; therefore, the standardized level of rabies clinics in a province or municipality was not consistent with the overall standardized level.

## Conclusions

Regional differences do exist in the distribution of rabies clinics in mainland China, with relative low numbers per million population in south and east China. The proportion of standardized rabies clinics is relatively low in mainland China. Efforts are still needed to improve rabies clinic standardization, especially regarding wound treatment and vaccination requirements. This study provides new strategies to improve rabies control by identifying the problematic areas in need of additional clinics. Future research should focus on the improvement of rabies clinics standardization.

## Data Availability

The databases were obtained from the Chinese Center for Disease Control and Prevention (http://www.chinacdc.cn) and the National Statistical Bureau (http://www.stats.gov.cn). However, public access to the databases is closed. The datasets are available from the corresponding author on reasonable request.

## Abbreviations

ABTC: Animal bite treatment center
CDC: The Chinese Center for Disease Control and Prevention
NDRIS: The National Disease Reporting Information System
PEP: Post-exposure prophylaxis
RIG: Rabies immunoglobulin
WHO: World Health Organization
